# Discovery, Synthesis, and Scale-up of Efficient Palladium Catalysts Useful for the Modification of Nucleosides and Heteroarenes

**DOI:** 10.3390/molecules25071645

**Published:** 2020-04-03

**Authors:** Shatrughn Bhilare, Harshita Shet, Yogesh S. Sanghvi, Anant R. Kapdi

**Affiliations:** 1 Department of Chemistry, Institute of Chemical Technology, Nathalal Parekh Road, Matunga, Mumbai 400019, India; shatru.3635@gmail.com; 2 Department of Chemistry, Institute of Chemical Technology-Indian Oil Odisha Campus, IIT Kharagpur Extension Centre, MouzaSamantpuri, Bhubaneswar 751013, Odisha, India; harshitashet8@gmail.com; 3 Rasayan Inc., 2802, Crystal Ridge Road, Encinitas, CA 92024-6615, USA; ysanghvi@rasayan.us

**Keywords:** nucleoside modification, palladium, catalysis, cross-coupling reactions, heterocycles, water-soluble catalyst

## Abstract

Nucleic acid derivatives are imperative biomolecules and are involved in life governing processes. The chemical modification of nucleic acid is a fascinating area for researchers due to the potential activity exhibited as antiviral and antitumor agents. In addition, these molecules are also of interest toward conducting useful biochemical, pharmaceutical, and mutagenic study. For accessing such synthetically useful structures and features, transition-metal catalyzed processes have been proven over the years to be an excellent tool for carrying out the various transformations with ease and under mild reaction conditions. Amidst various transition-metal catalyzed processes available for nucleoside modification, Pd-catalyzed cross-coupling reactions have proven to be perhaps the most efficient, successful, and broadly applicable reactions in both academia and industry. Pd-catalyzed C–C and C–heteroatom bond forming reactions have been widely used for the modification of the heterocyclic moiety in the nucleosides, although a single catalyst system that could address all the different requirements for nucleoside modifications isvery rare or non-existent. With this in mind, we present herein a review showcasing the recent developments and improvements from our research groups toward the development of Pd-catalyzed strategies including drug synthesis using a single efficient catalyst system for the modification of nucleosides and other heterocycles. The review also highlights the improvement in conditions or the yield of various bio-active nucleosides or commercial drugs possessing the nucleoside structural core. Scale ups wherever performed (up to 100 g) of molecules of commercial importance have also been disclosed.

## 1. Introduction

The molecules of special significanceand essential components in all living organisms as well as viruses are nucleic acids that are commonly involved in a wide range of cellular functions [1,2]. The basic role of nucleic acids is a promoter of processes such as replication, transmission, and transcription of genetic data [3,4]. Nucleotides are the building blocks of nucleic acids that are composed of heterocyclic aglycone, a glycoside unit, and a phosphate group [5],while the nucleoside unit lacks the presence of a phosphate group. Nucleic acid chemistry has over the past several decades shown its extensive presence in various fields including medicinal chemistry [6]. Through these studies, it has been found that any small change in the structure of nucleosides has a profound effect on their potential bioactivity [7]. Currently, there are more than 40 nucleoside analogs that have been approved as drugs and several others are in clinical trials [8]. It is for this reason that the modification of nucleosides has gained much attention from the scientific community in recent years [9]. Often, modifications are carried out on either the heterocyclic base or the sugar, and in certain cases, even on the phosphate group [10]. Such modified nucleoside analogs have found extensive utilities in the field of biology, biochemistry as biological probes, virology, cancer research, and pharmaceutical agents [11]. Some of the focused areas that have benefited immensely from nucleoside modifications are the discovery and development of antivirals [4,8], antitumor drugs [12], antisense oligonucleotides [13], mutagenic and DNA repair study [14], fluorescent nucleosides [15],C-nucleosides [16], DNA labeling [17,18], etc.

**Figure 1:**
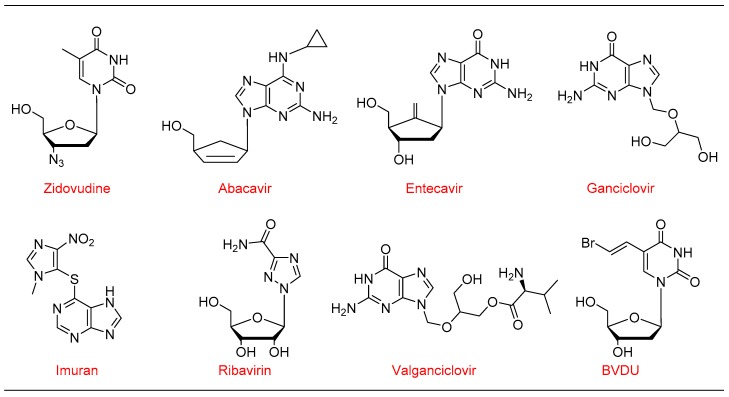
List of Food Drug Administration (FDA) approved modified nucleoside drug molecules.

One of the most promising approaches in the catalytic modification of substrates under mild conditions are transition-metal catalysis providing higher order of stereo-, regio-, and chemo-selectivity under a mild set of reaction conditions [19]. With the development of an efficient catalyst or in combination with activating ligand systems, transition-metal catalyzed processes have opened an efficient pathway for the construction of C–C and C–heteroatom (such as C–N, C–O, and C–S) bonds, which are commonly difficult under conventional synthetic conditions [20]. The transition metal-catalyzed processes have demonstrated a high level of productivity, sustainability, and practical industrial applications surpassing that of many traditional organic syntheses [21]. Furthermore, the use of transition-metal mediated processes allow the transformationsto be performed using green chemistry principles, helping to minimize the waste (E factor kept lower), and the possibility of recyclability and scale-up that would result in the development of cost effective and economical protocols. However, the main drawback of using transition metals in all of these transformations is the possible presence of higher amounts of metal content in the product, which is not acceptable for further applications and could be easily addressed by lowering the metal catalyst loading. The Lipshutz group recently reported catalytic protocols that were at the ppm level of the metal catalyst, thus minimizing the metal content in reaction and in turn offering an environmentally benign process [22]. Among all the transition metal catalyzed processes, palladium-based catalytic reactions have demonstrated the widest applications in the academic field as well as in industries [23,24]. Particularly, palladium-catalyzed cross-coupling reactions have proven to be important synthetic tools for tackling challenging synthetic problems associated with nucleoside transformations [25,26]. This has culminated into the development of a wide variety of synthetic approaches based on the involvement of palladium catalysts for accessing nucleoside-based drugs; a few of theseare listed in Figure 1 [27].

**Figure 2:**
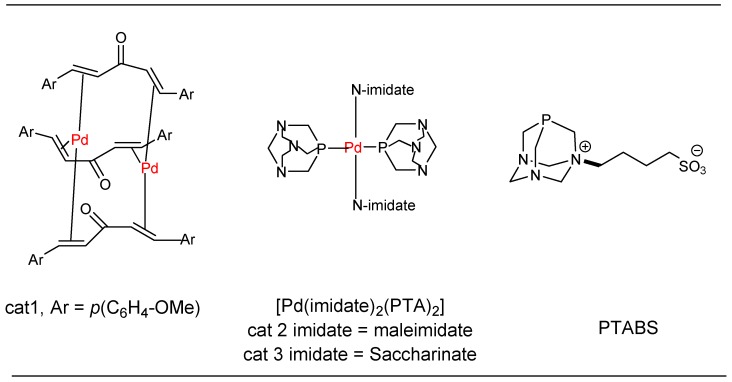
Systems for nucleoside modifications and cross-coupling.

One of the earliest examples of nucleoside modification using the Pd-catalyst was accomplished by Bergstrom in 1976 during the Heck reaction [28]. Subsequently, several protocols have been reported by varying Pd-precursors, ligand and coupling partners for the modification of nucleosides via Suzuki, Heck, Sonogashira, Stille, Negishi, Buchwald–Hartwig, and Tsuji–Trost reactions [29,30,31,32]. Recognizing the importance of Pd catalysis in the chemical modification of nucleosides, our group was inspired to develop sustainable (green) mild, efficient protocols with scalability at the forefront [18]. Many research groups in this area have reported a broad range of Pd-catalysts for performing the nucleoside modifications; however, the reported protocols suffer from several limitations such as the use of air and moisture sensitive catalytic systems (phosphines), high catalyst loading, volatile solvents, and use of toxic reagents [25]. These protocols are applicable for either purines or pyrimidines, and no single catalytic system is available for the modification of both types of nucleobases where a solitary Pd-based catalytic system could be utilized effectively [29]. The inherent water solubility of unprotected nucleosides, nucleotides, and oligonucleotides further motivated us to develop a water-soluble catalytic system that would be ideal for scale-up [33,34]. In this regard, we have successfully developed several Pd-based catalytic systems for the cross-coupling reactions of halonucleosides and chloroheteroarenes (Figure 2). Initial studies were directed toward the development of an efficient protocol for the Heck coupling of 5-iodo nucleoside using the Pd–dba catalyst [35]. Next, in order to have an efficient water-soluble catalytic system, we developed Pd–imidate based complexes in collaboration with the Serrano group, which were successfully utilized for Heck and Suzuki–Miyaura cross-coupling reactions [36,37,38]. Subsequently, we also developed triazaphosphaadamantane (PTA) based water-soluble phosphines, which are used in combination with palladium acetate for C–C and C–heteroatom bond forming reactions [39,40,41,42,43].

**Figure 3:**
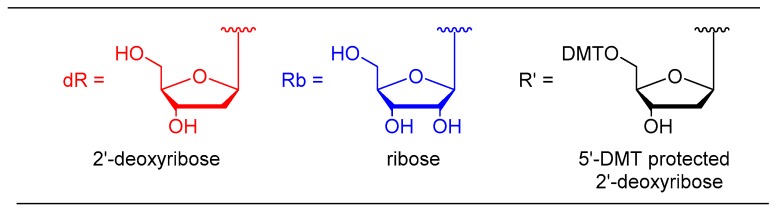
Structures abbreviation.

To minimize the duplication in structures, we will abbreviate the sugar units in forthcoming structures, as depicted in Figure 3.

### 1.1. Heck Alkenylation Using cat1

**Scheme 1:**
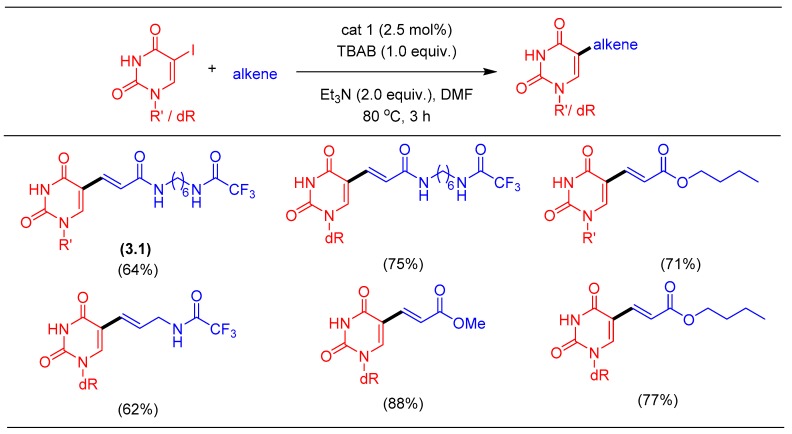
Pd-catalyzed Heck alkenylation of uracil derivatives.

For the demonstration of the Heck reaction, we elected the synthesis of pyrimidine modified nucleosides. For example, Ruth linker 3.1 is the molecule of interest due to its application in post-synthetic conjugation of oligonucleotides [44]. During the process of oligonucleotide synthesis, the free amino functional group (after the deprotection of trifluoro acetyl group) of the Ruth linker reacts with fluorophore or quencher to form the hybridized oligonucleotide probe. In this way, the free amino functionality is used for coupling with dyes and thus for labeling of DNA. Lyttle et al. reported on the synthesis of the Ruth linker as a reagent and method for the assembly of internally labeled DNA [45]. This protocol requires several steps in the synthesis of the target molecule, making it less attractive for scale-up. In our early studies, we employed a ligand-free system based on Pd_2_dba_3_by varying the electronics on the dba ligands. Fairlamb et al.’s work was our impetus for the non-innocent behavior of dba ligand and the ease of optimizing the Heck cross-coupling [46,47]. After screening the process parameters of the Heck reaction with 5-IdU such as temperature, base, catalyst, additive, etc., we developed the optimal reaction conditions as summarized in Scheme 1.

The phosphine-free catalytic system was further optimized to enable the column-free isolation of the coupled product as an easily isolable solid [35]. The Heck alkenylation protocol was also extended toward the synthesis of other cross-coupled products, as shown in Scheme 1. In this study, we demonstrated the synthesis of the Ruth linker on the 10 g (15.23 mmol) scale after appropriate process optimization. In comparison to the literature reports, which provided lower yields, our protocol was superior both in yield and ease of scale-up. Ruth linker synthesized by column-free procedure exhibited a ppb level of palladium confirmed by Inductively Coupled Plasma-Mass Spectrometry (ICP-MS).

The majority of cross-coupling reactions of nucleosides has been carried out in organic solvents and there is a need to move away from the use of environmentally detrimental volatile organic components (VOC) for industrial scale-up. Water is a useful alternative solvent for various chemical transformations because it is non-flammable, non-toxic, and renewable in nature [48]. More importantly, initial solubility of the unprotected nucleoside starting material in water allows the reaction to progress quickly and the insoluble hydrophobic product isolation is made easier by a simple filtration technique [49].

**Figure 4:**
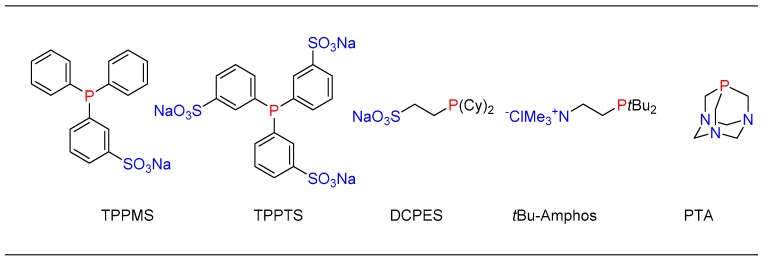
List of water-soluble phosphine ligands.

To further improve the utility of catalytic systems, a combination of metal precursors with a water-soluble ligand would assist the isolation process as the catalyst would have affinity toward the water phase, thus offering recyclability [50]. In this context, several water-soluble ligands have been reported for cross-coupling reactions (Figure 4) [33].

### 1.2. Suzuki-Miyaura Cross-Coupling Using cat 2

**Scheme 2:**
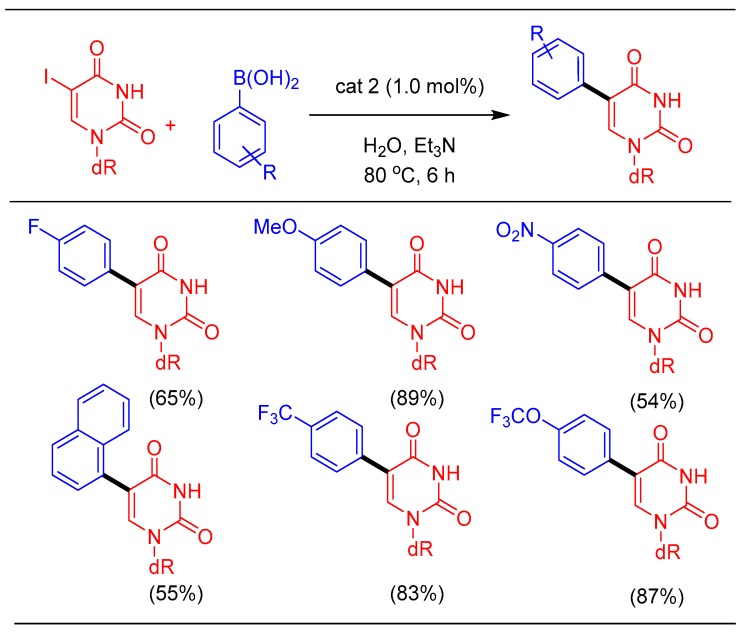
Suzuki–Miyuara reaction on 5-IdU.

After a careful survey of water-soluble ligands reported in the literature, we decided to explore and use the PTA ligand due to its unique properties such as small (atom-efficient) and basic structure, water-solubility, air stability, and the ability to bind more strongly to metal atoms compared to other bulky phosphine ligands [22]. In a collaborative effort with the Serrano group, PTA based palladium complexes were synthesized and screened for the catalytic cross-coupling of nucleoside in water. In this study, the first set of [Pd(imidate)_2_(PTA)_2_] were synthesized and found to exhibit appreciable water-solubility (110 mg/mL) [37,51]. The titled complexes can be easily synthesized by the reaction between *trans*-[Pd(imidate)_2_(SMe_2_)_2_] and PTA ligand. All the synthesized complexes were characterized by spectroscopic techniques as well as single-crystal x-ray analysis. The synthesized Pd-complexes were evaluated for Suzuki–Miyaura cross-coupling of 5-IdU. Amongst these complexes, [Pd(maleimidate)_2_(PTA)_2_] was found to be most active, although others work equally as well. With thorough process investigation of the reaction conditions for promoting Suzuki cross-coupling in water as a solvent, the coupling of 5-IdU with various aryl boronic acids provided good to excellent yields (Scheme 2) [38].

### 1.3. Heck Alkenylation Using cat 3

**Scheme 3:**
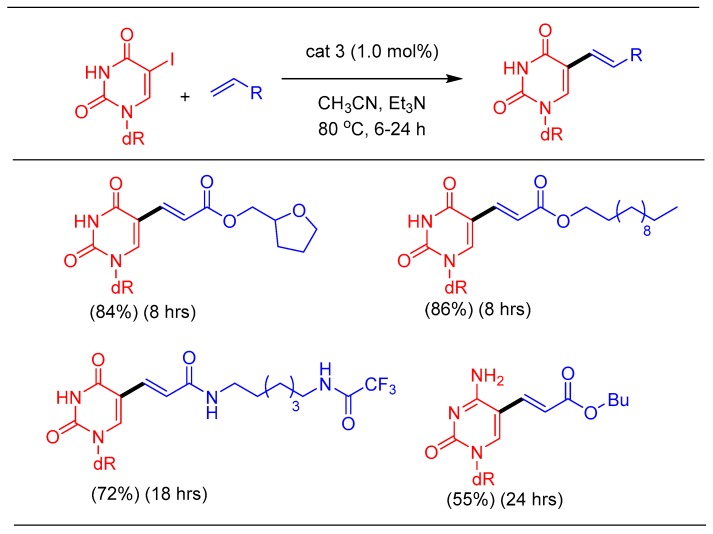
Heck alkenylation of 5-IdU using the Pd-imidate catalyst.

Pd-imidate catalysts were also screened for the Heck alkenylation reaction on unprotected nucleosides to generate novel structures for pharmaceuticals [27]. Optimization of process for the Heck reaction between 5-IdU and acrylate revealed the necessity of acetonitrile as the solvent, possibly due to the low solubility of the alkene counterpart. Using the optimal reaction conditions, nine different examples for the alkenylation of 2’-deoxyuridine analogs were obtained while the same conditions were also found to be useful in catalyzing Heck alkenylation on 5-iodo-2’-deoxycytidine (Scheme 3) [51].

### 1.4. Synthesis of Antiviral Drug Brivudine (BVDU) Using cat 3

**Scheme 4:**
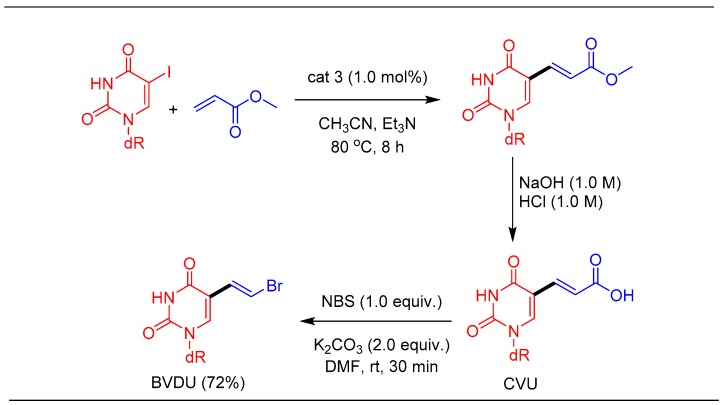
Synthesis of brivudine(BVDU) using the Pd-imidate catalyst.

Brivudine (BVDU) is an antiviral nucleoside analog used in the treatment of herpes zoster virus (HSV-1). Hervé et al. in 2014 reported the synthesis of BVDU using a Pd-catalyzed Heck alkenylation reaction in 56% yield by using 10 mol% of Pd(OAc)_2_ as a catalyst [52]. With a highly active catalytic system in hand, we explored the possibility of addressing the issues related to BVDU synthesis such as lower yields and the use of a high catalyst concentration. The Pd-imidate catalyst, due to its efficiency, was found to catalyze the Heck reaction at 1.0 mol% catalyst loading and the subsequent ester hydrolysis, followed by bromo-decarboxylation, provided BVDU in 72% overall yield (Scheme 4) [51].

In order to check the scalability of the improved protocol for the synthesis of BVDU, a scale-up of 10 mmol was performed with reproducible yield. To our delight, the new catalytic process permits recycling of the complex three-times without compromising the yield.

### 1.5. Suzuki–Miyaura cross Coupling on Four Natural Nucleosides Using cat 3

**Scheme 5:**
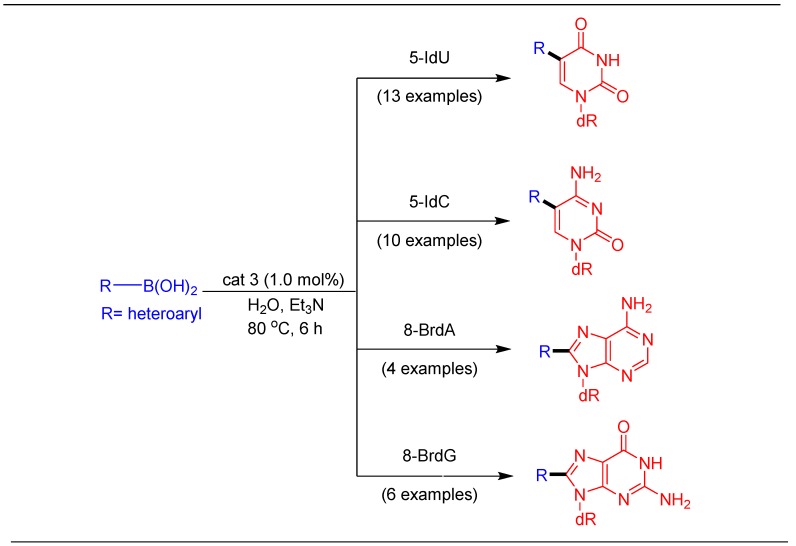
Suzuki–Miyaura arylation of nucleosides using a Pd-imidate catalyst.

Next, our objective of developing asingle catalytic system for the modification of all four natural nucleosides (dU, dC, dA, and dG) was investigated. In our previous report, we developed Suzuki–Miyaura coupling of 5-IdU using a [Pd(maleimidate)_2_(PTA)_2_] catalyst [51]. In order to establish a single and highly efficient catalytic system for both purine and pyrimidine nucleosides, the [Pd(saccharinate)_2_(PTA)_2_] catalyst emerged as a suitable candidate after careful screening of all the complexes under different process parameters (Scheme 5) [37]. This effort led to the synthesis of 33 modified nucleosides using a single catalytic system with potential for further scale-up.

**Scheme 6:**
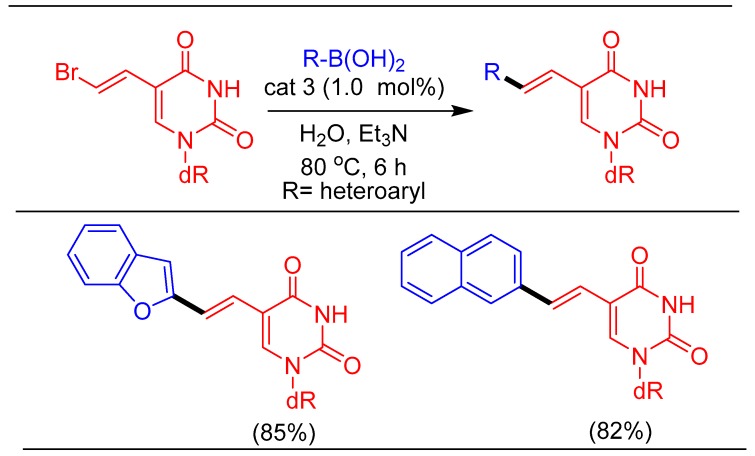
Synthesis of fluorescent nucleosides following Suzuki–Miyuara reaction of BVDU.

Synthesis of novel fluorescent molecules conjugated to nucleoside is an important area of research for biochemists and have found applications in gene detection, single nucleotide polymorphism (SNP) typing, and fluorescence imaging [53]. In order to synthesize extended fluorescent nucleoside analogs using our catalytic system, we took advantage of the reactive vinyl bromide moiety in BVDU and used it as a starting material. Further functionalization of BVDU following Suzuki–Miyaura cross-coupling provided excellent yields of the fluorescent nucleoside analogs never reported before (Scheme 6).

**Scheme 7:**
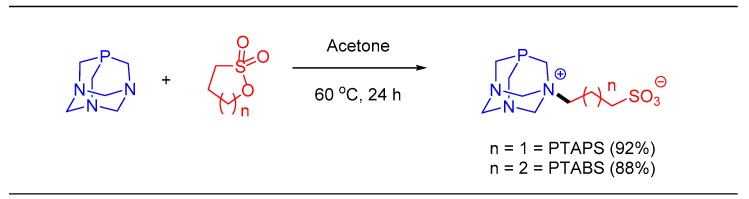
Synthesis of water-soluble triazaphosphaadamantane(PTA) based ligands.

Next, we explored the application of the water-soluble Pd-imidate complex for the nucleoside modifications beyond Suzuki and Heck reactions. Our attempts to perform the Sonogashira reaction with a Pd-imidate complex resulted in less efficient cross-coupling. To overcome this hurdle and to develop a universal catalytic system with higher water-solubility and reactivity, we envisaged the modification of the triazaphosphaadamantane (PTA) ligand as the possible solution. Alkylation of PTA generating an ionic character may offer enhanced solubility in water. In 2013, Kuhn et al. discussed imparting water-solubility in *N*-heterocyclic carbene (NHC) ligands by the introduction of SO_3_^-^ functionality [54]. Complexation with Ru and Os was further carried out to provide water-soluble NHC based complexes [55]. In a similar vein, our group also developed water-soluble NHC ligands and their Pd-based complexes. We used this to catalyze the Suzuki–Miyaura coupling in water and tested the anticancer activities of the products [56]. The introduction of sulfonate functionality in the NHC ligand was achieved by the ring opening of a cyclic sultone derivative by an amine or nucleophilic nitrogen atoms. Therefore, we hypothesized that the introduction of a sulfonate group on PTA may enhance not only the water-solubility, but also influence the catalytic efficiency. These considerations allowed us to synthesize a new class of water-soluble, PTA based ligands. In brief, PTA was allowed to react with 1,3-propane sultone and 1,4-butane sultone separately, providing PTAPS and PTABS ligands, respectively (PS is propane sulfonate and BS is butane sulfonate), in high yields as shown in Scheme 7 [39].The newly synthesized ligands were fully characterized by various spectroscopic techniques and single crystal x-ray analysis.

### 1.6. Suzuki–Miyaura cross Coupling Using Pd/PTABS

**Scheme 8:**
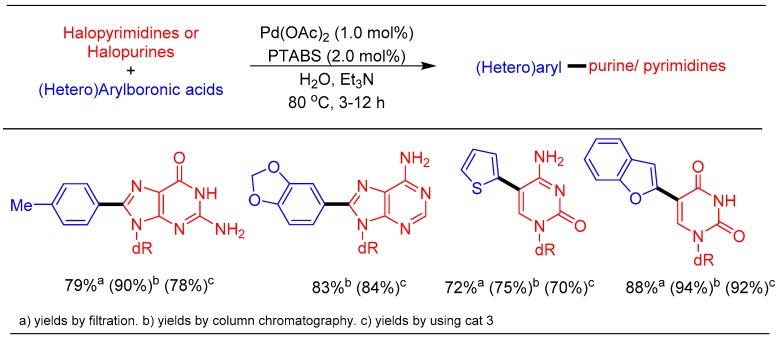
Suzuki–Miyuara reaction of halo-nucleosides using the Pd/PTABS catalytic system.

Application of PTABS and PTAPS as water-soluble ligands in combination with palladium acetate was first tested with the Suzuki–Miyaura cross-coupling of 5-IdU with different boronic acids in water as the solvent. Gratifyingly, PTABS proved to be an excellent ligand, amenable for coupling of a wide variety of boronic acids with halo-purine and pyrimidine nucleosides (Scheme 8) [39].

To the best of our knowledge, high yields of the cross-coupled products with a single catalytic system and isolation of base-modified nucleosides via simple filtration was accomplished for the first time. In comparison, the yields obtained with the Pd/PTABS catalytic system were significantly improved over the same reactions carried out with Pd-imidate complex ([Pd(imidate)_2_(PTA)_2_]). The high solubility and excellent reactivity of the Pd/PTABS system was further exploited for a chromatography-free process, which is an important attribute for scale-up. However,another trait is the ability to recycle the catalyst, thus demonstrating the “greenness” of the process. We are pleased to report that the Pd/PTABS system allowed an efficient cross-coupling of 5-IdU with benzofuran boronic acid furnishing non-chromatographic isolation and reuse of the catalyst for 8 consecutive cycles without any appreciable loss in the yield (Scheme 8). These results clearly paved the path for further exploration of Pd/PTABS system for other cross-coupling reactions and their scale-up.

### 1.7. Sonogashira Cross-Coupling Using Pd/PTABS

**Scheme 9:**
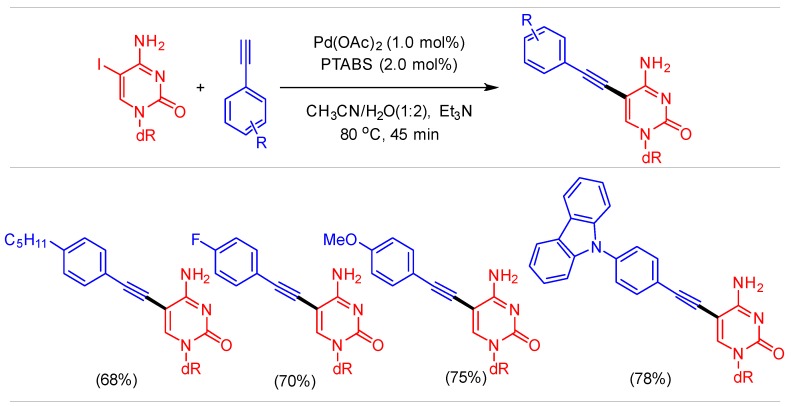
Cu-free Sonogashira reaction of 5-IdC.

One of the shortcomings of ([Pd(imidate)_2_(PTA)_2_]) system was the execution of Sonogashira coupling with halo-nucleosides. Therefore, we investigated the Sonogashira coupling of 5-IdC with substituted alkynes and the resultant outcome was promising showing our ability to perform Cu-free Sonogashira in 45 min (Scheme 9). The short reaction time and low catalyst loading sets this protocol apart from other conventional Sonogashira reactions [57].

**Scheme 10:**
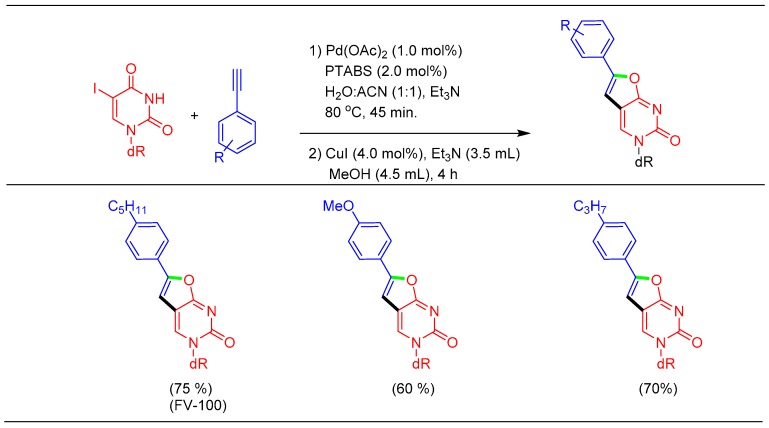
Tandem Sonogashira/cyclization strategy for the synthesis of BCNA.

Sonogashira cross-coupling of 5-IdU with an alkyne derivative was found to be highly efficient followed by in situ cyclization to furnish the bicyclic product. Cyclization of the Sonogashira product of 5-IdU has been reported for the synthesis of the bicyclic nucleoside analogs (BCNA) [58]. The BCNA analogs exhibited potent clinical VZV activity in the picomolar range whereas being non-toxic even at micromolar concentrations [58]. To access these therapeutic scaffolds, it was envisaged to employ tandem catalytic reactions involving a sequential Sonogashira reaction, followed by a cyclization reaction to be performed in the same pot, without the isolation of the intermediate alkyne product, making the overall process atom economic and helps to avoid an additional purification step [59]. In this regard, after completion of the Sonogashira reaction, we allowed Cu-catalyzed cyclization to afford the corresponding BCNA in higher yields when compared to that reported in the literature (Scheme 10). The employment of 5-pentylphenyl acetylene as the coupling partner offers the product known as FV-100 (Cf-1743), which is a nucleoside-based antiviral drug under phase III clinical trial [60].

**Figure 5:**
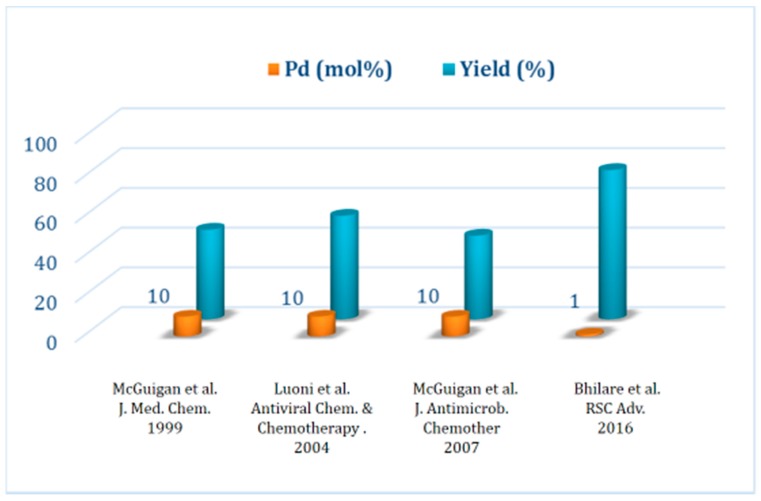
Summaryof the FV-100 synthesis protocols.

The true impact of the process efficiency of the Pd/PTABS catalytic system is summarized in a graphical manner showing a comparison with other protocols available in the literature. The protocol developed in our lab clearly allows for the significant reduction of the catalytic loading of palladium (10 mol% down to 1.0 mol%), making the process attractive for commercial manufacturing (Figure 5) [39,58,61,62]. This study was the cornerstone for us to further explore the utility of a tandem one-pot reaction with a Pd/PTABS catalytic system. 

**Scheme 11:**
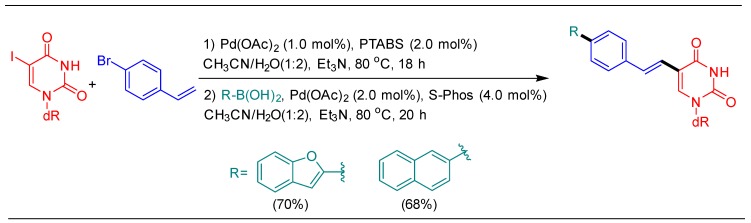
Tandem one-pot Heck/Suzuki coupling of 5-IdU.

To illustrate the utility of the tandem protocol, first the Heck coupling of 5-IdU with bromostyrene was performed, installing a bromo handle for further functionalization. The product underwent Suzuki–Miyaura cross-coupling in the second step, thus furnishing fluorescent nucleoside analogs possessing extended conjugation. These molecules serve as excellent building blocks for DNA diagnostic applications (Scheme 11) [39].

**Figure 6:**
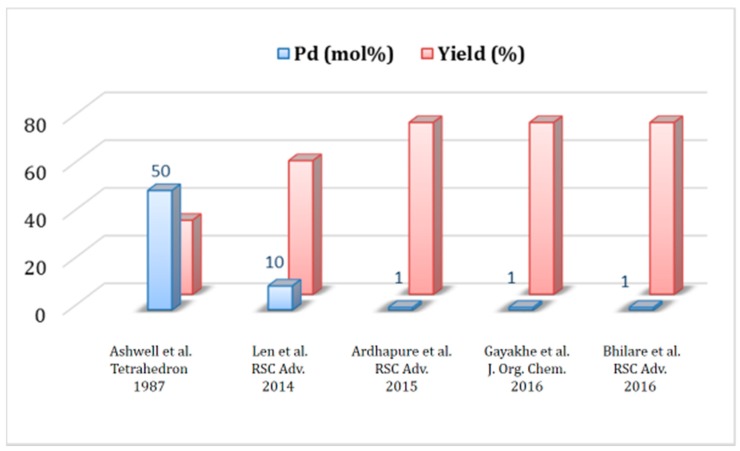
Comparative chart for the synthesis of BVDU.

Additionally, the Pd/PTABS system was also successfully implemented for the synthesis of BVDU in similar yield compared to [Pd(imidate)_2_(PTA)_2_]. The synthesis of BVDU was achieved using the same synthetic steps as that depicted in Scheme 8, except for the change in catalyst to the Pd/PTABS system. A summary chart shows the process superiority of our protocol, offering an alternative for large-scale production of the antiviral drug (Figure 6) [37,39,51,52,63].

The foregoing discussion allowed Heck, Suzuki, and Sonogashira reactions to be carried out for the modification of nucleosides using the [Pd(imidate)_2_(PTA)_2_] and Pd/PTABS system. As nucleosides and their analogs are of immense interest to the industry, a broader perspective would be to employ the new catalytic systems to address issues related to heteroarene functionalization. This could be related to the construction of C–C or C–heteroatom (C–N, C–O, C–S) bond formation between heteroarenes and nucleophiles where only a limited number of examples have beenreported in the literature that are applicable to nucleosides. The following sections describe our recent endeavors demonstrating additional examples involving the functionalization of heteroarenes including nucleosides as well as molecules of industrial importance.

### 1.8. Amination Reaction Using Pd/PTABS

**Scheme 12:**
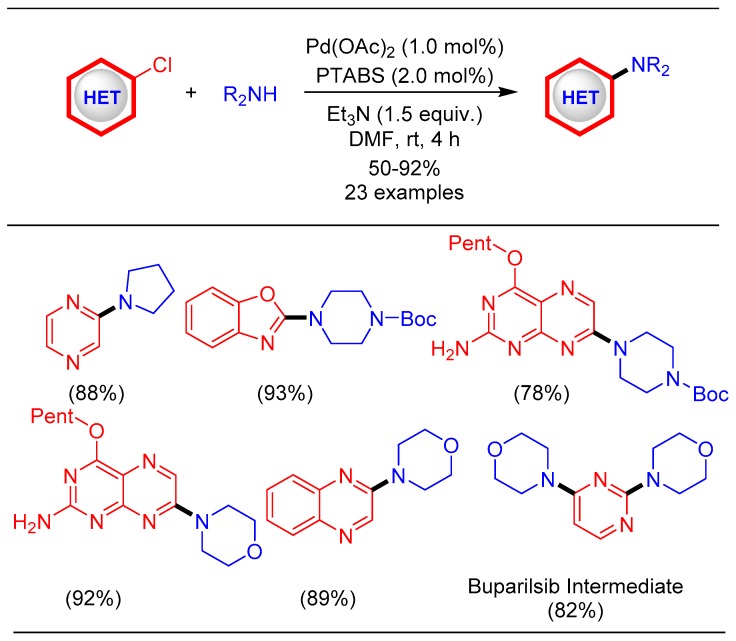
Pd/PTABS catalyzed amination of chloroheteroarenes.

Initially, it was decided to study the amination of heteroarenes as the amine functionality is present in many important biomolecules that are involved in life governing processes as well as pharmaceutical drugs. Palladium-catalyzed amination is an attractive strategy for the C–N bond forming reaction with several research groups reporting different Pd-based catalysts for the amination reactions [64,65]. Most of these protocols, however, suffer from several major drawbacks such as the high temperature of the reaction, longer reaction time, and low substrate scope of heteroarenes, which make the developed protocols synthetically less attractive [66]. These limitations were successfully addressed through the development of a room temperature amination protocol of chloroheteroarenes using the Pd/PTABS catalytic system (Scheme 12) [42]. Using the optimal reaction conditions for the amination of chloroheteroarenes, a wide range of heteroarenes were efficiently coupled with secondary amines. This work provided easy access to molecules such as Buparlisib intermediate (Scheme 12). Buparlisib is an important drug candidate exhibiting pan-PI3K inhibitor activity that is under clinical development, especially for brain tumor treatment [67].

**Scheme 13:**
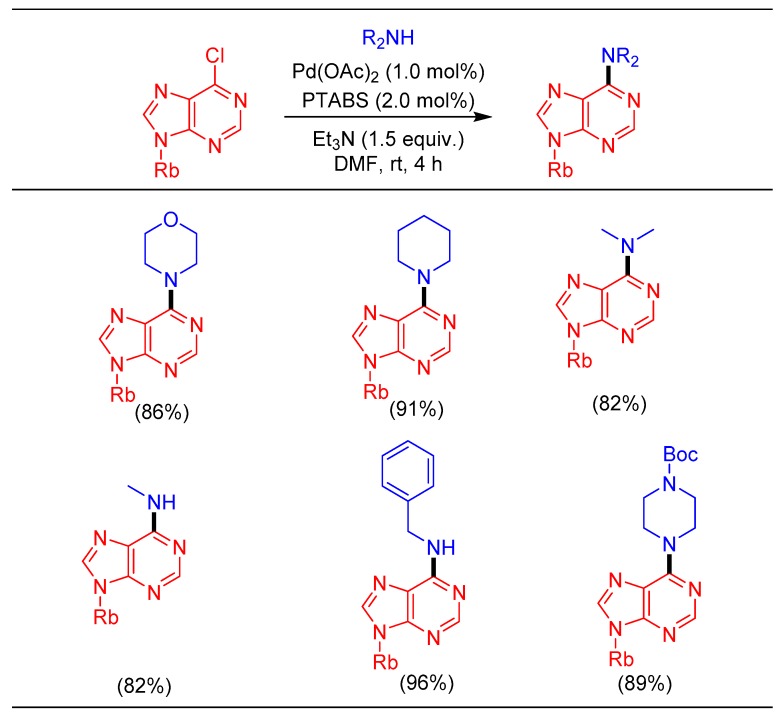
Pd/PTABS catalyzed amination of purine analogs.

This protocol also offered significant improvement in the yield over the previously reported methods for the amination of 6-chloropurine riboside providing 6-*N*-substituted adenosine analogues exhibiting promising applications in pharmacology and biochemistry (Scheme 13) [42].

**Scheme 14:**
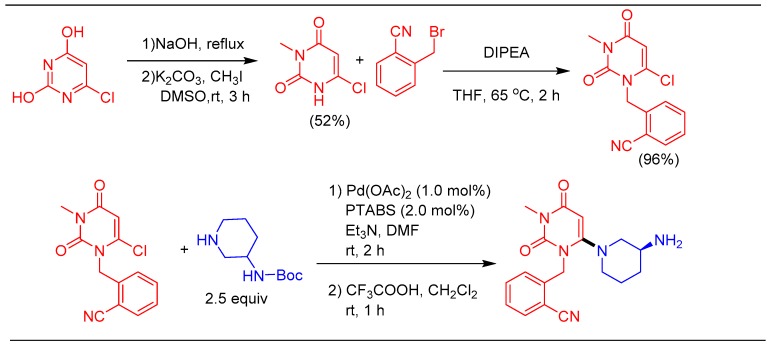
Formal synthesis of alogliptin using the Pd/PTABS catalytic system.

Our goal will also be to implement catalytic protocols for industry relevant productsand in lightof this,a formal synthesis of uracil-based orally administered anti-diabetic drug alogliptin was undertaken(Scheme 14) [42,43]. The synthetic scheme required the conversion of 6-chloropyrimidine-2,4-diol to 2-((6-chloro-3-methyl-2,4-dioxo-3,4-dihydropyrimidin-1(2*H*)-yl)methyl)benzonitrile in three steps.

Subsequently, Pd/PTABS catalyzed amination and deprotection of the N–Boc group offers the desired product in 92% yield. The developed strategy avoids the conventional approach based on the nucleophilic substitution method and allows alogliptin synthesis under relatively milder reaction conditions, offering improved yield [42,68].

### 1.9. Etherification Reaction Using Pd/PTABS

**Scheme 15:**
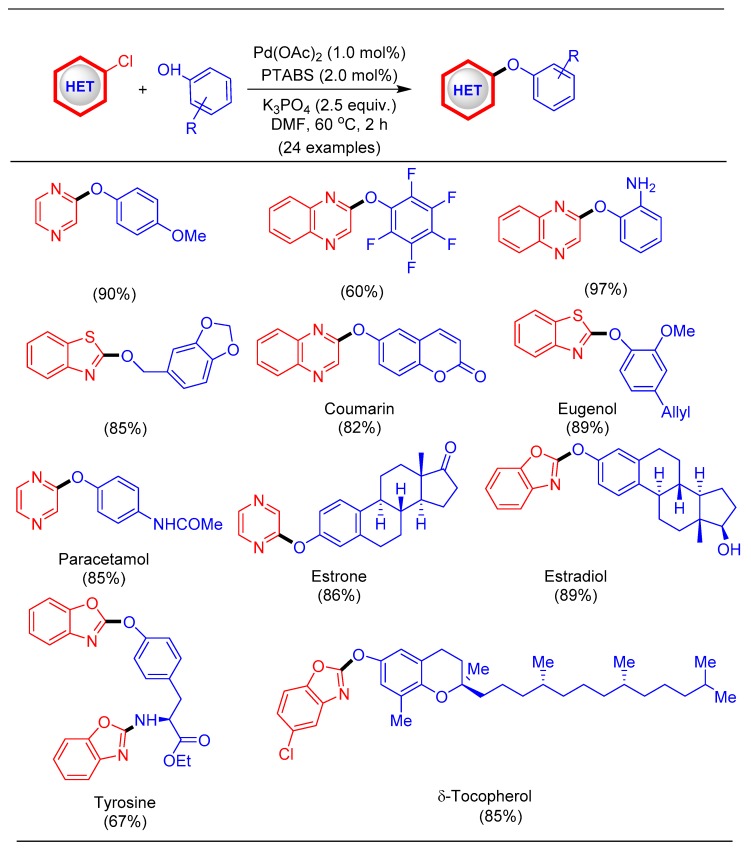
Pd/PTABS catalyzed etherification of chloroheteroarenes.

Inspired by these results for the amination of chloroheteroarenes, we further decided to explore the reactivity of the Pd/PTABS catalytic system for C–O and C–S bond forming reactions of chloroheteroarenes. Like an amine, ether functionality is also commonly present in many natural products, drugs, and pharmaceuticals witha heteroaryl skeleton [69]. Transition-metal catalyzed etherification reactions that are reported by several researchers are commonly related to copper-catalyzed Ullmann and Chan−Lam−Evans reactions as well as Pd-catalyzed etherification [70]. However, many of these protocols suffer from problems such as the use of a stoichiometric amount of catalyst, low substrate scope, harsh reaction conditions (such as higher temperature and stronger base), and moderate yields [71]. To address these shortcomings, we developed an efficient, milder protocol for the etherification of chloroheteroarenes [40]. Optimization of reaction conditions and substrate scope was performed under relatively low temperature conditions furnishing good yields withvisibly no influence of electronics on the phenol coupling partner. The substrates were not limited to simple phenols, but were extended to alcohols and heteroaryl phenol as well as bioactive phenols, which could be potential drug candidates and scaffolds for medicinal chemistry (Scheme 15) [40].

**Scheme 16:**
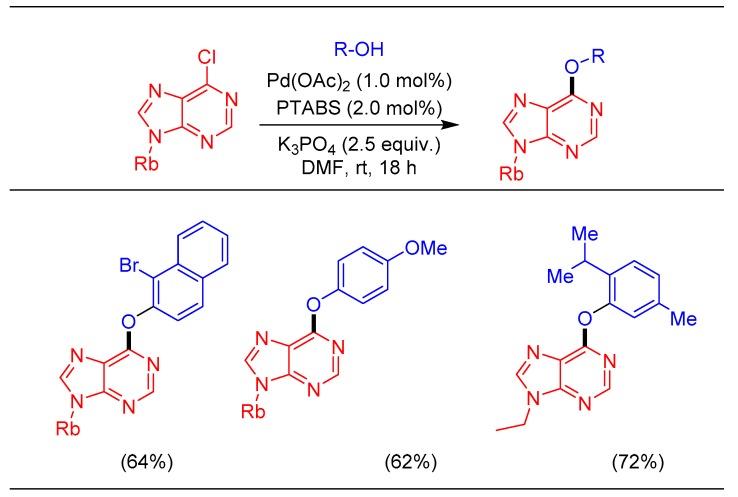
Pd/PTABS catalyzed etherification of purines.

Modified purine nucleosides, particularly the C-6 substituted (by C-, N-, O-, S-) analogs, are a well-studied class of bioactive molecules [72]. The traditional synthetic approach for the construction of this structural core is executed through classical nucleophilic substitution reactions. Often, the electron poor nucleophiles providelower yields and require higher temperatures and longer reaction times [73]. Our process development efforts led to an efficient strategy of etherification, achieving synthesis of C-6 aryloxy substituted purine derivatives in good yields using the Pd/PTABS catalytic system (Scheme 16). Execution of room temperature etherification of nucleosides is an attractive feature for the transformation of sensitive molecules, which was executed effectively using the developed catalytic protocol.

**Scheme 17:**
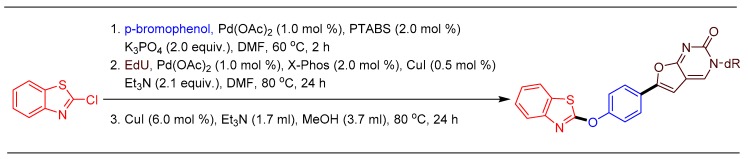
Triple tandem one-pot reaction for the synthesis of BCNA analogs.

One of the salient features of the Pd/PTABS catalytic system was the chemo-selective coupling of the C–Cl vs. C–Br bond that provided a functional handle for subsequent tandem catalytic reaction. We demonstrated the synthesis of new fluorescent nucleoside analogs (BCNA) via unique triple tandem catalytic reaction involving the sequence etherification/Sonogashira/cyclization disclosed herein (Scheme 17) [39,58,74].

**Scheme 18:**
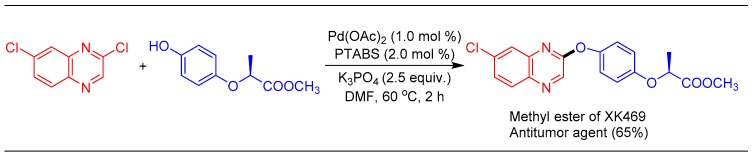
Synthesis of XK469 using the Pd/PTABS catalytic system.

To showcase the industrial utility and mildness of the etherification reaction, the synthesis of XK-469 (an antitumor agent) [75] was achieved in 65% yield by employing the Pd/PTABS catalytic system (Scheme 18). Our strategy is milder and less time consuming compared to the previously reported protocols that are based on nucleophilic substitution reactions [76].

### 1.10. Thioetherification Reaction Using Pd/PTABS

**Scheme 19:**
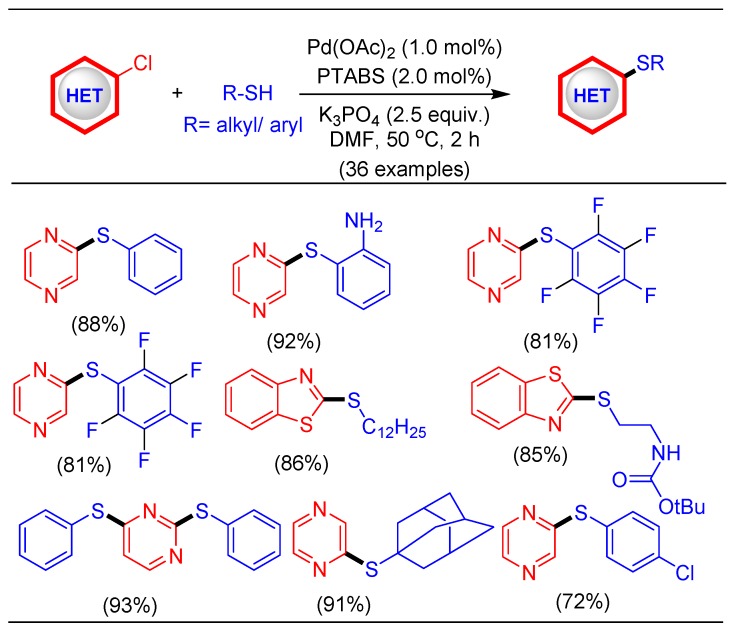
Pd/PTABS catalyzed thioetherification of chloroheteroarenes.

Stoked by the excellent results for C–N and C–O coupling using the Pd/PTABS catalytic system, we decided to next explore the efficacy of the new catalytic system for C–S bond forming reactions [77]. Thioether linkages are present in numerous scaffolds exhibiting biological significance. Additionally, thioethers are commonly employed as key building-blocks for the introduction of functionalities such as sulfone, sulfoximine, and sulfoxide. These reasons were the main thrust points for the process development of efficient protocols enabling thioetherification [78]. The traditional synthetic approaches of the substitution reaction lack the functional group tolerance, resulting in the formation of appreciable amounts of undesired products [79]. The role of transition-metal mediated processes, particularly Pd-catalyzed reactions, are looked upon as a promising alternative to these traditional approaches. However, the Pd-mediated reported processes for the C–S coupling reaction could only be facilitated at high temperature under a longer reaction time and with the use of a strong base [80,81,82]. The main challenge, therefore,indesigningaPd-catalytic system toenablethioetherification is also to prevent catalyst poisoning (by coordination of sulfide with a metal atom) [83,84] or the formation of disulfide as a side product [85]. Appropriate process optimization of the reaction conditions and establishing awide substrate scope (36 examples), we recently reported lowtemperaturethioetherification employing a catalytic Pd/PTABS system (Scheme 19) [41]. The gentle catalytic reaction makes this procedure attractive for preparative scale commercial products.

**Scheme 20:**
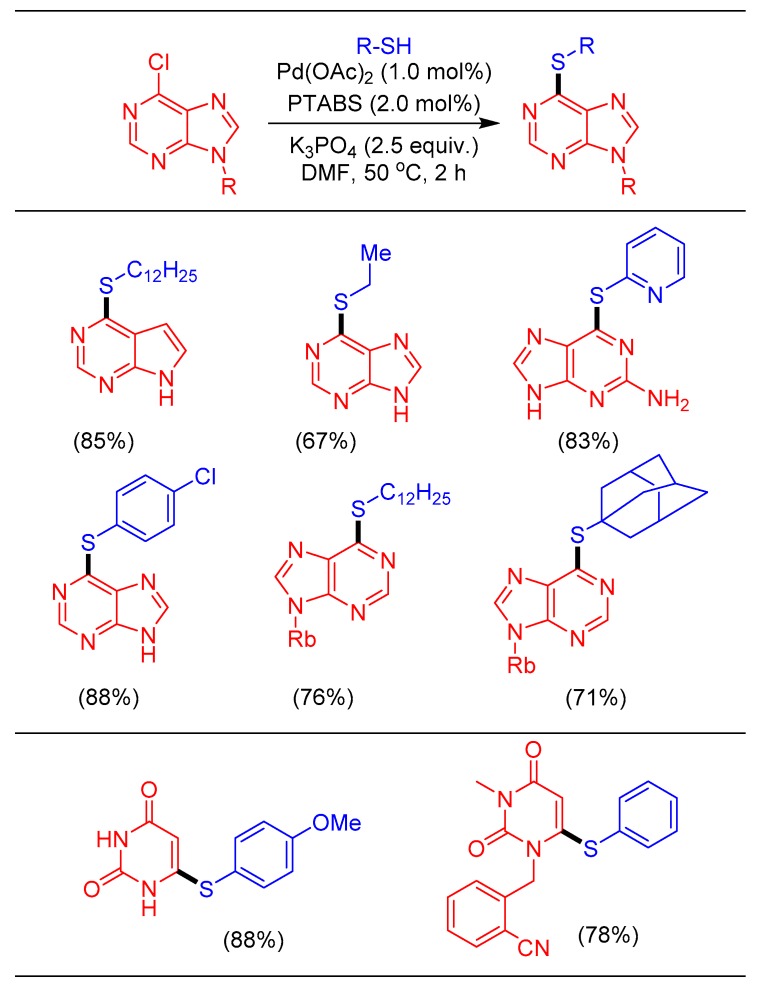
Pd/PTABS catalyzed thioetherification of purine and pyrimidine.

These advances were utilized for the thioetherification of purine and pyrimidine structural motifs due to their importance as cytotoxic and immunosuppressive agents [86] as well as used in cancer treatment [87]. Synthesis of these thiolated molecules proceeded efficiently with a variety of alkyl and aryl thiols in good to excellent yields (Scheme 20).

**Scheme 21:**
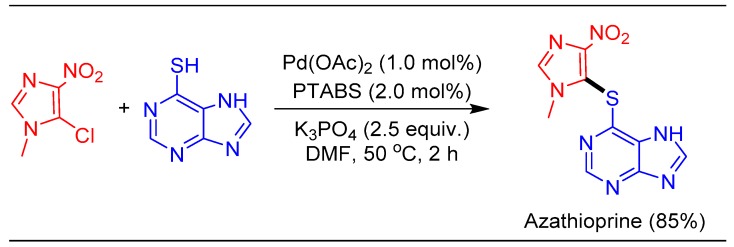
Pd/PTABS catalyzed synthesis of azathioprine.

The common theme of our research is to transfer the learning to a product of commercial interest. Therefore, utility of the efficient Pd/PTABS catalytic system was examined for the synthesis of an immuno-suppressive drug Imuran (azathioprine) [88]. Thioetherification of 6-mercaptopurine with 5-chloro-4-nitro-*N*-methylimidazole furnished azathioprine in 85% isolated yield (Scheme 21).

### 1.11. Aminocarbonylation Reaction Using Pd/PTABS

Next, we focused on the aminocarbonylation process using the Pd/PTABS catalytic system. Discovery of the CO gas as a reactant [89] has allowed several synthetic procedures and reactions to be reported that are using CO as a *C1* building block. Commercial scale processes such as Fischer–Tropsch, Otto Roelen, and Monsanto acetic acid are a few elite examples of this class [90]. CO gas also finds major application in the construction of industrially useful molecules having carbonyl functionality in their core structure [91]. The revolutionary utilization of CO gas using the Pd-catalyst was first initiated by Heck in 1970 [92]. The ease of incorporation of different nucleophiles leading to a wide variety of synthetically relevant products has certainly allowed carbonylation to be a well appreciated process in academia and industry [93].

**Scheme 22:**
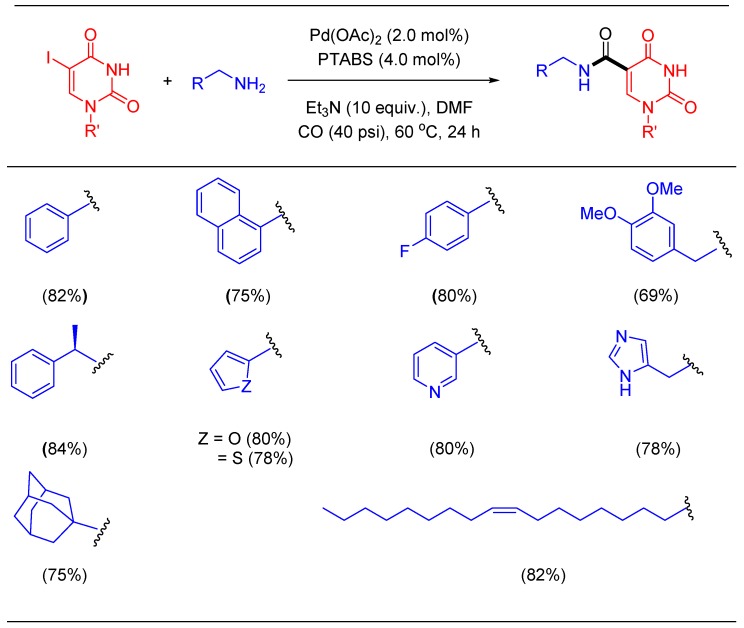
Pd/PTABS catalyzed aminocarbonylation of nucleoside.

Amide functionality that can be easily installed using aminocarbonylation has been utilized in many nucleoside scaffolds including synthesis of aptamers [94,95]. The amide group is also known to be useful for the stabilization of DNA as it provides an extra site to accept and donate H-bonding targeting protein interactions [96,97]. Thus, it can serve as an important tool for the in-vitro selection of protein-binding aptamers (SELEX process) [98] and for post-SELEX optimization of selected aptamers [99]. However, only a handful of protocols in the literature are available to construct an amide group on nucleosides,therefore, inspiring us to evaluate the new catalytic system for the amidation of nucleosides [29,96]. Extensive screening of process conditions to couple 5′-*O*-(4,4′-dimethoxytrityl)-5-iodo-2′-deoxyuridine (5′-*O*-DMT-5-IdU) with benzylamine using CO gas as a *C1* source was carried out in a systematic manner. Pleasantly, we discovered that the Pd/PTABS system worked efficiently to install an amide group in protected 2′-deoxynucleoside using40 psi pressure of CO gas and a 60 °C reaction temperature. Clearly, this protocol is mild enough for carbonylation of DMT-protected molecules, offering easy access to modified nucleosides otherwise difficult to synthesize following literature protocols. The versatility of this process was proven via the introduction of a library of amide functionalities onto the core nucleoside structure (Scheme 22) [100]. Furthermore, this protocol was successfully scaled up to 10 g using naphthalen-1-ylmethanamine and the Pd/PTABS catalytic system.

**Scheme 23:**
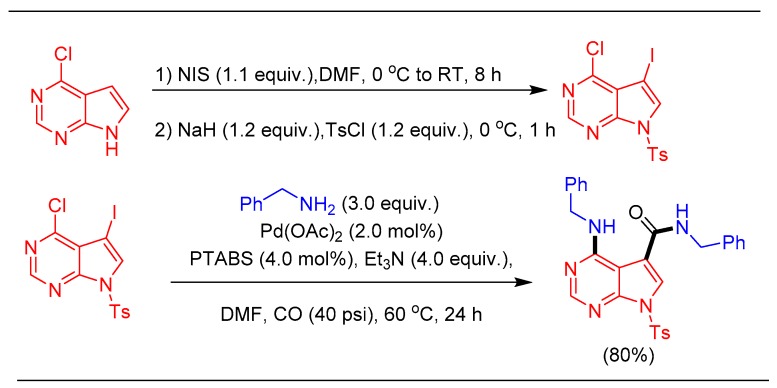
Pd/PTABS catalyzed synthesis of thesangivamycin precursor.

Further demonstration of the synthetic potential of the catalytic strategy was made possible by successfully performing a one-pot amination/amidationstrategyon6-chloro-7-iodo-7-deaza purine to provide the Sangivamycin precursor in 80% isolated yield (Scheme 23) [101].

**Scheme 24:**
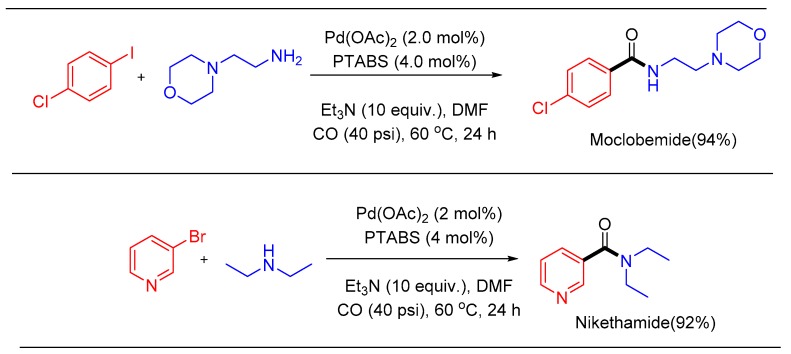
Pd/PTABS catalyzed synthesis of moclobemide and nikethamide.

To showcase the utility of the new process and implementation for obtaining commercial products, the synthesis of two drug candidates, moclobemide [102] and nikethamide [103], was accomplished using the Pd/PTABS catalytic system in excellent yields (Scheme 24) [100].

### 1.12. C─H Bond Functionalization of 1,3,4-oxadiazoles Using Pd/PTABS

**Scheme 25:**
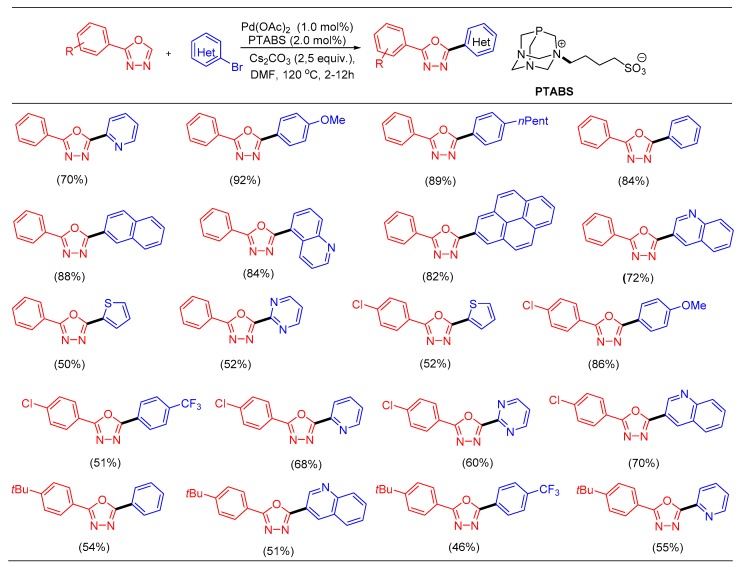
C─H bond functionalization of 1,3,4-oxadiazoles using (hetero)aryl iodides.

Our recent contribution to the growing field of C─H bond functionalization of heteroarenes reports the functionalization of 1,3,4-oxadiazoles with a variety of (hetero)aryl bromidesusingthe Pd/PTABS catalytic system. A large number of bromo(hetero)arenes were employed with most providing good to excellent yields of the C─H arylated products (Scheme 25) [104]. 

The(hetero)arylationstrategy for oxadiazoles also provided access to a commercially available fluorescent organic scintillation material, butyl-PBD as well as anti-tubercular agent PHOXPY in good yields. Mildness of the protocol and its compatibility to incorporate other catalytic systems in combination with it such as the Sonogashira reaction (steroidal substructures bearing alkynes), Heck alkenylation, or Suzuki–Miyaura coupling in a one-pot tandem procedurecould further be useful in the development of potential drug candidates given the biological relevance of modified 1,3,4-oxadiazoles.

### 1.13. Scale-Up of Ruth Linker Using Pd/PTABS

We believe that the catalytic process improvements described herein are poised for implementation in the industry. One such example is the large-scale synthesis of Ruth linker being currently practiced at Sapala Organics [105]. Ruth linker is a modified 2′-deoxyuridine analog, reported, and used by several research groups for the post-synthesis conjugation of diagnostic oligonucleotides with a fluorescent tag or an affinity probe. 

**Figure 7:**
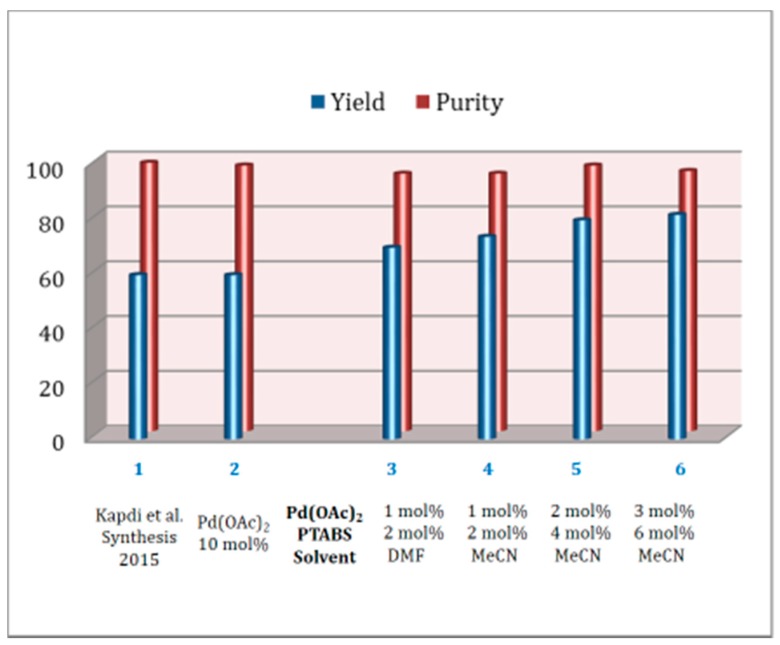
Improvement in the synthesis of Ruth linker.

The Heck alkenylation protocol for the synthesis of a Ruth linker engaging Pd/PTABS catalytic system was fully optimized in the following manner. The centralfocus for the process development of this protocol was to avoid the use of an additive, isolate the product without chromatography, reduce the concentration of the catalyst, and improve the overall yield. Initial protocol for the synthesis of Ruth linker at Sapala Organics utilized 10 mol% of Pd loading, offering 60% yield of the desired product (entry 2, Figure 7). This protocol was fully optimized where impact of solvent, catalyst ratio, temperature, and time were carefully studied (entries 3–6, Figure 7). Among the various solvents tested, acetonitrile allowed the formation of the desired product in the highest yield. For optimum yield, the ratio of Pd:PTABS was 2:4, where Ruth linker was obtained in 80% isolated yield and 97% purity (determined by HPLC). Increasing the catalyst concentration did improve the yield, but the purity was compromised (entry 6, Figure 7).

**Scheme 26:**
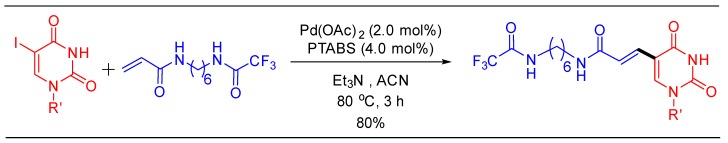
Pd/PTABS catalyzed synthesis of Ruth linker.

The final scale up of the reaction was performed on 100 gmandthe protocol was also found to be reproducible on a large-scale (Scheme 26). More importantly, the final isolation of the high purity product was accomplished without column chromatography. It is noteworthy that both an acid labile DMT group and base labile TFA groups survive the reaction conditions demonstrating the neutral character of the catalytic system.

Given the widespread applications of the Pd/PTABS catalytic system described in this article, it was necessary to establish a commercial supplier of the PTABS ligand [106]. We are pleased to state that Strem Chemicals has taken appropriate steps to offer PTABS as a standard catalog product (catalog no. 15-5715, CAS no. 1430837-91-4, IUPAC name: 3,5-Diaza-1-azonia-7-phosphatricyclo[3.3.1.13,7]decane, 1-(4-sulfobutyl)-, inner salt).

## 2. Conclusions

In conclusion, we developed catalytic systems for the modification of nucleosides to offer sustainable and efficient protocols. The developed water-soluble catalytic systems have been successfully used for the synthesis of chemically modified nucleosides via the Suzuki–Miyaura, Heck, Sonogashira, Buchwald–Hartwig amination, etherification, and thioetherification as well as aminocarbonylation reactions. Remarkably, the catalytic process is more efficient, milder (tolerant to variety of groups), and sustainable whencompared to the literature protocols with the possible recyclability of the catalytic system in a few cases. The improved strategies have been applied for the synthesis of several molecules of commercial interest such as BVDU, FV100, Ruth linker, alogliptin, XK469, azathioprine, nikethamide, and moclobemide drug molecules as well as buparlisib and sangivamycin synthetic precursors. These protocols have been found to be highly efficient with respect to yields and maintaining low Pd-loading for a good part of the studies. Interestingly, large numbers of novel nucleoside structures have been assembled in a short period of time following the new catalytic system. We are making efforts to automate these protocols, allowing high throughput library synthesis of novel chemical entities (NCE) for pharmaceutical drug discovery companies. The process chemistry advances described in this review are expected to provide a firm platform and framework, leading to catalytic reactions with reduced environmental impact. In summary, the Pd/PTABS catalytic system is broadly applicable for a variety of cross-coupling reactions, offering an excellent resource for process chemists in both academia and industry.

## Abbreviations

FDA	Food and Drug Administration
PTA	Triazaphosphaadamantane
PTABS	Triazaphosphaadamantane butane sulfonate
PTAPS	Triazaphosphaadamantane propane sulfonate
ICP-MS	Inductively Coupled Plasma Mass spectrometry
BVDU	Brivudine
CVU	Carboxyvinyl2’-deoxy uridine
